# Vicarious contrast media excretion: the bloody effusion Doppelgänger

**DOI:** 10.31744/einstein_journal/2025AI1383

**Published:** 2025-10-03

**Authors:** Danilo de Oliveira Santana Ramos, André Vaz, Eduardo Kaiser Ururahy Nunes Fonseca

**Affiliations:** 1 Universidade de São Paulo Faculdade de Medicina Hospital das Clínicas São Paulo SP Brazil Hospital das Clínicas, Faculdade de Medicina, Universidade de São Paulo, São Paulo, SP, Brazil.; 2 Universidade de São Paulo Faculdade de Medicina Hospital das Clínicas São Paulo SP Brazil Instituto do Coração, Hospital das Clínicas, Faculdade de Medicina, Universidade de São Paulo, São Paulo, SP, Brazil.

**Figure 1:**
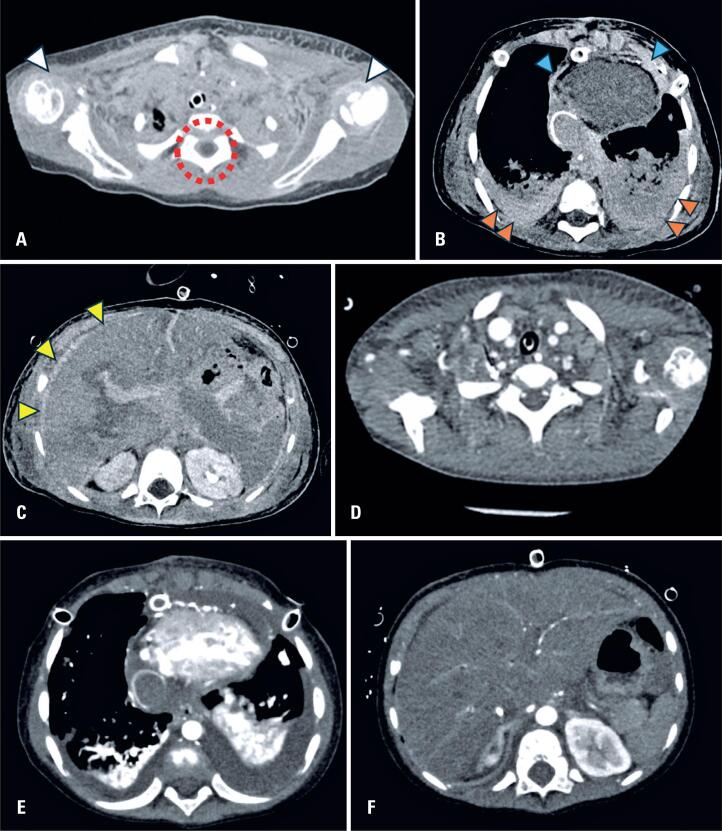
Non-contrasted chest and head computed tomography (CT) scan of a 3-year-old male, showing vicariant contrast media excretion 4 h after an angiographic evaluation (A, B, and C). (A) Spontaneous hyperdensity in the subcutaneous tissue, muscles, joints, including synovial fluid (white arrowheads) and cerebrospinal fluid (red dashed circle) at the cervicothoracic junction. (B) Spontaneous hyperdensity of the pleura (orange arrowheads), and pericardium (blue arrowheads) at the inferior third of the chest. (C) Spontaneously hyperdense ascites (yellow arrowheads) at the thoracoabdominal transition. Ten days later, these findings were no longer observed in a contrast-enhanced chest CT scan (D, E, and F)

A 3-year-old boy was admitted to the hospital with decompensated congenital heart disease, hypotension (serum lactate: 100mg/dL), and acute renal failure (serum creatinine: 3.54mg/dL). Hemodynamic angiography was performed to assess cardiac pressures with a volume of 120 mL at a rate of 15 mL/s of iodinated contrast. Approximately 4 h later, the patient underwent a chest computed tomography (CT) scan using a 320-section scanner without intravenous injection of iodinated contrast or use of general anesthesia. Vicariant contrast excretion was observed in virtually all the body tissues (Figure 1). Ten days later, after further evaluation using chest CT, these findings were no longer observed (Figure 1).

The use of contrast media is well-established as a critical component in diagnostic and interventional radiology. Among the various types, iodine-based contrast media are most commonly used for diagnostic and therapeutic purposes, including CT evaluation, interventional procedures, and hemodynamics. These agents may be administered through several routes: intravenous, rectal, oral, or directly into the fistulous tract or biliary tree.

Typically, iodinated contrast media are primarily excreted through renal filtration, with minimal excretion via the biliary and gastrointestinal tracts.^(1,2)^ Under normal physiological conditions, virtually no contrast medium is detected a few hours after administration, even at high doses. However, persistent contrast medium may be detected in diverse body tissues hours after its intravenous administration.

Understanding the vicarious excretion of iodinated contrast medium is crucial for accurate radiological interpretation and clinical decision-making. This often-overlooked phenomenon can significantly affect imaging results, potentially mimicking serious conditions such as abdominal hemorrhage, hemothorax, hemopericardium, and intracranial hemorrhage. This mimicry can lead to misdiagnosis and inappropriate treatment. Radiologists across all subspecialties must be aware of this phenomenon to differentiate it from genuine hemorrhagic manifestations and ensure accurate diagnosis and optimal patient care.
